# Corruption and the Other(s): Scope of Superordinate Identity Matters for Corruption Permissibility

**DOI:** 10.1371/journal.pone.0144542

**Published:** 2015-12-09

**Authors:** Anne C. Pisor, Michael Gurven

**Affiliations:** Department of Anthropology, University of California, Santa Barbara, Santa Barbara, CA, United States of America; University of Maribor, SLOVENIA

## Abstract

The decision to engage in corruption—public and private corruption, nepotism, and embezzlement—is often attributed to rational actors maximizing benefits to themselves. However, the importance of reciprocal relationships in humans suggests that an actor may weigh the costs of harms of her corrupt behavior to individuals who may generate future benefits for her. We hypothesize that actors who have a larger circle of actual and potential social partners will have more individuals to consider when generating harms and will thus be less likely to find corrupt acts permissible than actors with smaller circles of valued others. Using data from the World Values Survey and European Values Study (WVS), we explore whether participants with a larger geographic identity or a greater number of group memberships (i.e. a larger scope of actual and potential social partners) are less likely to find accepting bribes permissible. We find mixed support for our hypotheses, but consistently find that WVS participants with local, country, continent, or world geographic identities are less likely to find accepting a bribe permissible than those with regional identities—that is, actors whose primary identities that encompass more than their region find corruption less permissible. We discuss the importance of considering an actor’s valuation of others when modeling corruption persistence, noting that establishing scopes of positive valuation is a precursor to predicting where actors will target benefits and shunt costs.

## Introduction

Corruption, commonly defined as taking advantage of public office for private gain [1,2], is considered a major deterrent to economic development [3]. The World Economic Forum [4] estimates that 5% of global GDP is lost to corruption each year. Pervasive corruption is also blamed for reducing public and private trust [5,6] and for promoting greater socioeconomic inequality [7,8]. The prevalence of corruption (or its perceived prevalence, as is often measured) is higher in countries with non-democratic institutions, strong kin ties, and greater ethno-linguistic heterogeneity (measured as the number of ethnic groups or languages spoken in a nation; [1,9,10]). Individuals perceive corruption as more prevalent when transparency is low (e.g. [11]), often true in contexts of high income inequality [12].

Whether the focus is on country-level or individual-level analyses, this research holds that rational actors will engage in corrupt behaviors when the expected benefits outweigh the expected costs. However, corrupt acts generate harms for others, and most models of corruption assume that actors are unconcerned about those who will have to absorb any costs the actors generate. Alternatively, one might expect an actor to be less willing to engage in a corrupt act if it will harm bystanders that she positively values as actual or potential social partners (i.e. individuals with whom she currently engages, or could potentially engage in repeated, mutually-beneficial interactions). The rational logic of avoiding corrupt behavior when the immediate benefits might seem high is that generating harms for others may reduce the likelihood of later being selected as a trustworthy social partner.

This paper explores how having larger numbers of positively valued social partners affects an actor’s likelihood of finding corruption permissible. We hypothesize that an actor who identifies with larger superordinate groups, where superordinate groups unify sub-groups (e.g., religious groups, ethnic groups) of both known individuals and strangers who are positively valued as potential social partners [13,14], will be more likely to avoid and disapprove of corrupt behavior because she stands to harm a greater number of potential social partners, thereby potentially damaging her reputation. Both harm to others and reputational damage will reduce the likelihood that other superordinate group members will trade with, support, or buffer the actor in the future. If the sum total of these costs outweighs the selfish benefits of engaging in corrupt acts, an actor may avoid them altogether. We test this hypothesis using individual judgments of corruption permissibility from the World Values Survey and European Values Study, as judgments of corruption permissibility may reflect one’s own willingness to engage in corrupt acts. If superordinate group size predicts corruption permissibility, this relationship may explain why countries with high kin loyalties, e.g. low-income or predominantly Catholic countries, are perceived by their citizens to be more corrupt than high-income, Protestant countries with fewer kin loyalties [1,15], and why subjective ethno-linguistic heterogeneity (i.e. whether an actor views local groups as diverse and different from one another) is a better predictor of corruption prevalence than objective ethno-linguistic heterogeneity [16–20]. It is less a question of religious affiliation or number of ethnic groups in a country as it is the number of people an actor values sufficiently to avoid generating costs for them. Our results show that superordinate group size, as proxied by the geographic size of one’s primary group identity, explains variation in corruption permissibility not considered by previous research. This relationship suggests an avenue for future investigation. Further, we outline how approaching the question of corruption persistence from an evolutionary perspective motivates the present hypothesis and can organize some existing findings.

### Corruption as extended self-interest

A broad definition of corruption is the private appropriation of a public resource, which generates costs to society. Narrower definitions depict corruption as an abuse of power by public officials (“public” corruption). Researchers espousing a narrow definition believe broader interpretations ought to distinguish private corruption (e.g. bribery of a non-public figure who can provide a service), nepotism (e.g. favoring kin or friends for a particular position or windfall), and embezzlement (e.g. drawing extra money from an organization while in a power position) from public corruption [21]. All four contexts—public and private corruption, embezzlement, and nepotism—have features in common, however. First, in all four, actors strategically channel benefits to self, kin, or social partners. Indeed, actors are known to use corrupt acts to build and maintain social relationships [22–24], obtain resources [25–27], and avoid undesirable costs [28,29]. Second, in all four, an actor's behavior violates national or international laws or norms. Third, all diminish societal well-being because private gain comes at the expense of others, either by increasing the cost of a service (whether in monetary or other currency, including time) or by drawing from a depletable resource (such as public funds). Given these commonalities, consistent with some existing work [22,30] we broadly define corruption as behaviors providing private gain to self, kin, and/or other group members that 1) generate harms for others by 2) “jumping the queue” of resource allocation [1] 3) in violation of norms or laws that *may* be applied to the local context, e.g. international laws that may or may not be adopted in practice. We say “may” because the perceived wrongness of corrupt acts may not track the magnitude of the social costs generated [22,25,31] and corruption may be construed as a gift or an earned right by bystanders [32,33].

A rational actor may engage in a corrupt behavior, or find a corrupt behavior permissible, when its perceived net benefits outweigh those of lawful or norm-consistent behavior. However, what are the relevant benefits and costs? The standard approach recognizes biases in corrupt behavior that favor kin and social partners (nepotism and cronyism; e.g. [3]), but the origin of these preferences is usually not examined [34]. Instead, these biases have been explained as reflecting ease of recruiting family member co-conspirators and lower likelihood of detection by authorities [35,36]. This exclusive focus on gains for an individual actor conflates self-interest with an actor’s preferences to support kin and maintain reciprocal relationships [37]. Preferences to support kin may be a feature of an evolved human psychology that generates benefits for self and kin, optimizing biological fitness even if generating costs for non-kin [38,39].

Favoring group members can also directly benefit an actor’s fitness by enabling others to return those favors via reciprocity [40,41]. One reason to favor group members is the need to buffer risk and uncertainty in resource access, though additional motivations may include gaining information access, political clout, or mates. The unpredictability of food resources is a defining characteristic of traditional human subsistence strategies that requires buffering amongst individuals to reduce food insecurity [42–45]. Even with the introduction of market-related options for risk management (e.g. insurance, savings, government-funded disaster relief), investing in and harvesting one’s networks of information and exchange remains crucial [46–48]. An actor’s access to potential social partners, and the resources they provide, can be damaged by the generation of harms, especially if these costs can be traced to the actor herself [49]. In both small-scale [50] and large-scale societies [51], sharing partners and allies are at a premium [52]. Actors with reputations for in-group stinginess [45] or who generate harms are not preferred as social partners [53,54]. An actor need not explicitly consider the consequences of her behavior for valued others, but only behave *as if* she were considering both her own welfare and theirs [55]. Her other-regarding behavior may be steered by psychological mechanisms favored by natural selection, including emotions such as shame and pride [56] and the ability to empathize with others’ emotions [57]. Increased exposure to out-group members (e.g., via globalization) could also increase preferences to exhibit fairness toward a larger number of individuals (cosmopolitan attitudes [58]). Our predictions apply with equal force to actors with cosmopolitan attitudes: when an actor can form partnerships with a larger number of people (e.g., when she is integrated in a social network), she may feel more empathy toward these individuals [59] and cooperate more with them [58,60]. In sum, we should expect people to be sensitive to costs and benefits that affect their own fitness interests (or proxies for fitness, such as wealth and status) and that of their kin, actual and potential social partners [61,62].

The individuals whom an actor positively values as potential social partners, which include strangers, are likely to be those with whom she identifies. Having a superordinate group identity (e.g., identifying with one’s region or nation [63], or even the globe [59]) increases the likelihood that an actor values members of groups that are otherwise out-groups for her, such as ethnic groups or religious groups. Indeed, even in small-scale societies, the potential to reap valuable gains from trade, coalition formation, and risk management often transcends the otherwise high transaction costs of interactions between ethnic and religious groups [64,65]. We thus treat the scope of an actor’s superordinate identity as a proxy for the scope of individuals whom she considers to be potential social partners.

As aforementioned, those who cannot locally access needed resources may seek more contact with a larger scope of individuals than those who already have access [65–67]. However, this logic may not extend to individuals in the most dire of situations: those with the lowest incomes and the poorest health may have a short time horizon and be less likely to weigh the well-being of out-group members heavily. The opportunity cost of lost partnerships only increases in situations of resource scarcity (e.g. due to a lack of government institutions), as individuals place an even higher premium on reliable social partners [68].

We suggest that the larger an actor’s scope of positively valued individuals, the more people’s welfare she will have to weigh before generating harms they must absorb; in turn, the larger the scope of her positive valuation, the less likely she will be to find corruption permissible. In this paper, we test whether proxies for a larger scope of positive valuation—the size of one’s primary geographic identity and the number of groups to which one belongs—can account for variation in corruption permissibility above and beyond that captured by more traditional predictors of corruption from the literature. We explore these predictions using individual-level data from the World Values Survey and European Values Study (hereafter, WVS refers to both). While country-level data are frequently used in research on corruption, country-level corruption indices are not necessarily good predictors of individual-level behavior [69,70]. Further, by employing a large cross-national data set, we gain additional insight into propensity to engage in corruption across a variety of social, political, and economic contexts. We operationalize corruption as someone accepting a bribe in the course of their duties, a potential proxy for a participant’s own willingness to accept a bribe. Existing studies have shown that a number of country-level variables are correlated with country-level corruption, including objective religious and ethnic heterogeneity, economic inequality, and political freedom. We control for these factors, as well as country-level corruption itself (as measured by the perceptions of businesspeople and academics), as the context within which individual actors decide whether or not a corrupt act is permissible. Because confidence in the police and civil services, belief in God, household dependency (as number of children), level of education, and participant age and sex are uncorrelated with superordinate group size yet could affect a participant’s motivation to condone corruption, we include these variables in all models. We explore the role of a lack of resource access (as proxied by a summary measure of household income, satisfaction with finances, and subjective health), as lack of access may increase the potential benefits of resource access via additional network connections.

### Hypotheses and predictions

We test the hypothesis **(H1)** that an actor who positively values a larger group of people as actual or potential social partners (i.e. has a larger superordinate group) will be less likely to find corrupt acts permissible. Though we cannot determine who a participant envisioned as affected by these harms when deciding whether it was permissible to accept a bribe, we suggest that all else equal, permissibility will decline as superordinate group size increases. We operationalize this as follows:

**(P1.1)** Participants whose primary identity is at a larger geographic scale will find corruption to be less justifiable than participants whose identity is more local in scale.
**(P1.2)** Participants who belong to more civic, social and activity groups will find corruption to be less justifiable than participants with fewer memberships in these groups.

**(H2)** Corruption permissibility should be lower among those for whom the net benefits from additional partnerships are greater. We predict that:

**(P2)** Participants who stand to gain benefits from additional resource access (i.e. who do not have high levels of subjective or objectively measured income, or good health) will be less likely to find corruption permissible.

## Methods

To explore the relationship between a participant’s superordinate group size and corruption permissibility, we use data from the World Values Survey (www.worldvaluessurvey.org) and European Values Study (www.europeanvaluesstudy.eu). We control for variables important in the corruption literature that may constrain the availability or benefits of additional social partnerships, or that may influence corruption permissibility via paths other than those hypothesized here, using individual-level data from WVS and country-level data compiled from the United Nations, the World Bank, Freedom House, and Transparency International.

### Corruption Permissibility

To gauge corruption permissibility, we adopted a measure of bribery permissibility from the WVS. The present paper uses WVS data from 91 countries collected between 1981 and 2009 (n = 399,376). Analyses are limited to the subsamples for which observations were available on all variables; sample sizes (ranging from 5785 to 80,390) are reported with model estimates.

To gauge whether participants find corruption permissible, we employ a WVS question asking participants to indicate whether "someone accepting a bribe in the course of their duties" would always, sometimes, or never be justified. Participants’ responses were recorded on a ten point Likert scale, with 1 indicating that accepting a bribe was never justifiable. Because 74% of participants responded that corruption was never justifiable, to increase statistical power we collapsed all other responses into a single category, creating a binary (0 = "never," 1 = "at least sometimes") measure of corruption permissibility.

### Superordinate Group Size: Primary Geographic Identity and Number of Group Memberships

We adopt two measures of superordinate group size from the WVS. The first addresses participants’ primary geographic identity. This was a forced choice question: participants were asked, "To which of these geographic groups would you say you belong first of all?" They were allowed to choose between five levels of scale: local, region, country, continent, and world. In the full WVS sample, the majority picked "local" (41%) or "country” (34%) while only 10% each chose "continent" or "world". Because such a small number selected “continent” or “world,” we combined the two into a single category. The concept of local, regional, and country level identity was translated similarly in all surveys, however "continent" was not consistently translated in different areas of the world. In South America, for example, participants were asked whether they saw themselves as members of Mercosur or the Latin American community, depending on the country.

Our second measure of superordinate group size is a summary measure of group memberships. We counted the number of organizations to which a survey participant belonged (similar to [71]). Participants were asked about membership in 15 different groups, including local political movements, human rights movements, sports clubs, and religious groups. Only 45% of participants who responded belonged to any of those groups, which suggests a limitation in the use of these questions: there are likely organizations other than political, athletic, religious, and human rights groups to which participants belong. Very few individuals belonged to a large number of groups; to avoid the influence of outliers we capped the number of group memberships at two standard deviations above the mean (4.31). Further, because of differences in questions asked in different countries and across different years, only 133,711 of the 399,376 participants who responded to the bribery permissibility question answered questions about group membership, limiting the sample size for models that include this measure. We ran models including number of group memberships and models including primary geographic identity separately because of this sample size limitation.

### Resource Access: A Summary Measure

Per our predictions, other variables that may affect corruption permissibility include income, satisfaction with household finances, and subjective health, as a decline in each may increase the magnitude of benefits to be gained via risk buffering. We include both objective income and subjective financial situation because purchasing power and perceived need may be independent predictors of corrupt behavior. Income is an integer rank from 1–11 that is country-specific, such that participants with 11 have among the highest incomes in a country. Subjective income, how a participant compares her household income to what she wishes to earn, can provide additional information about a participant's perceived shortfalls. Participants were asked whether they were satisfied with their household finances on a scale of 1–10, with 10 as most satisfied. Participants rated their subjective health on a scale of 1–5, with 5 representing "very good" health.

### Individual-Level Controls

We control for other individual-level variables that may affect corruption permissibility, independently of having a larger superordinate group. Three variables identified by previous research on corruption are included, as well as four potential confounders. We control for belief in God using the same question that Atkinson and Bourratt [72] found to predict corruption acceptance in a WVS sample; they suggest that belief in a vigilant, omniscient God can help enforce cooperative behavior, a hypothesis supported by priming research [73,74]. Previous work has demonstrated that the functioning of government services, particularly the (bribe-free) enforcement provided by police, increases the likelihood of detection and punishment for corrupt acts. In our analysis, we combined the two questions probing confidence in two government services that can curb corruption, police and in civil services, into one summary variable: we reverse coded these variables so that confidence was measured on a scale from 1–4, where 1 was "[no confidence] at all" and 4 was "a great deal of confidence." We then centered the summary variable at zero. Several studies using WVS data have suggested that women are less corrupt than men [75] because "women will be less likely to sacrifice the common good for personal (material) gain" [76]. Men also tend to be less generous in Dictator Games, economic experiments that tap participant altruism and fairness [77,78]. We control for sex in our analyses accordingly. In all models, we control for age, education, and household dependency. Age may affect corruption permissibility independently of cohort, education, and employment effects [79]. Education increases exposure to a larger scope of individuals, but depending on the context of exposure, valuation for these individuals can be either boosted or lowered. Level of education was measured using a country-specific rank: participants with the lowest educational attainment in the country were coded as 1, while those with the highest were coded as 3. Number of children is a proxy for household dependency, which may diminish the effect of increased income on bribery permissibility. The distribution of number of children was negatively skewed, so we rounded all participants above 2 SD of the mean, 5.29 children, down to 5.29. Finally, we include the population size of a participant’s town as a robusticity check. The population of a participant’s town is an integer scale, with 1 being a town with a population of 2,000 or less and 8 being a population of 500,000 or more. Because town population was unavailable for many participants, we used this variable only as a check to avoid limiting sample size.

In an effort to situate one of our results, we also conducted exploratory analyses of the predictors of having a regional geographic identity. As we suggested in the Introduction, the relationship between kin biases and corruption permissibility [1,15] may be a function of an actor’s scope of valuation; as such, we explored whether a participant’s geographic identity (here, regional identity) was a correlate of their kin biases. As a proxy for kin biases, we include a question asking participants whether “more emphasis on family life” would be a good, neutral, or bad change to their way of life. We check whether profit motive is related to a regional identity, as profit motive has explained tolerance of corrupt behavior in some laboratory experiments (e.g. [80]). As a proxy for profit motive, we include whether participants believe “less emphasis on money and material possessions” would be a good, neutral, or bad change to their way of life. Finally, we examine whether participants with regional identities value more respect for authority figures, as respect for authority may decrease a participant’s likelihood of engaging in acts that harm others [81] and may be correlated with a regional identity, as those who identify with their region may be marginalized members of a nation and less bound to the national government [82]. As a proxy for respect for authorities, we include whether participants believe “greater respect for authority” would be a good, neutral, or bad change to their way of life.

### Country-Level Variables

Models including country-level variables are reported in the Supporting Information (S1 and S2 Tables; S1 and S3 Figs), as are the methods for these variables (S1 Appendix). We employed United Nations census data as a measure of objective, country-level religious and ethnic heterogeneity; World Bank measures of country population size, population density, and Gini coefficient; Freedom House’s Freedom in the World Political Rights Index; and Transparency International’s Corruption Perceptions Index. We also adopted the World Bank’s classification of countries into world regions, which we include in models reported in the main text.

### Statistical Methods

All models were fit using the R statistical package version 3.1.3 [83]. Using logistic regressions with logit links, we regressed corruption permissibility on the two hypothesized predictors of interest (primary geographic identity, number of group memberships), individual-level variables (income, satisfaction with household finance, subjective health, education, belief in God, confidence in police and civil services, age, and sex), and, in models reported in the Supporting Information (S1 and S2 Tables; S1 and S3 Figs), country-level variables (religious and ethnic fractionalization or polarization, population size and density, Gini, the FIW political rights index, and the CPI).

To evaluate whether the inclusion of proxies for superordinate group size improved model fit relative to models including only controls, we compared models using Akaike weights. The Akaike Information Criterion (AIC) is a goodness of fit measure that maximizes the likelihood of model fit while penalizing additional parameters. We weighted AIC values from several candidate models to identify the best fit model [84] using qpcR [85].

We predicted that participants whose resource access is low, but not too low, would stand to gain the most from a larger circle of potential social partners. We created a single summary variable to represent the resource access proxies: objective income, subjective income, and subjective health. Principal components analysis on standardized values suggested that, relative to subjective income, subjective health had a loading of 0.88 and objective income a loading of 0.94 on the first component. For the group membership subset, the loadings for subjective health and objective income were 0.82 and 0.98 that of subjective income, respectively. Accordingly, we standardized the three variables and summed them, weighting them according to their relative loadings in each subset. We then inverted the summary measures such that higher values indicate less resource access.

### Robusticity Checks

The countries in this data set are not a representative world sample. There are a number of ways to control for potential clustering of residuals driven by the particular set of countries sampled, two of which we employ here. To avoid collinearity issues, both modeling approaches first required that we exclude country-level variables from the model. Because country-level variables had constrained the sample size, the number of observations in models excluding country-level variables was four times the size that of models including country-level variables. We first ran multilevel models by including random intercepts for each country in our models (i.e. using logistic mixed-effect models implemented in lme4; [86]). Second, we fit the same models by including fixed effect country dummies in place of country random effects. Third, to check for any effect of shared cultural history, we fit fixed effect and random effect models controlling for world region in place of country. All model types provided consistent results for the variables of interest (with the exception of the region models for number of group memberships, as discussed below). Because of the four orders of magnitude increase in sample size when country-level variables were excluded, we focus on fixed effect models with country dummy variables in the text. Models including country-level variables are reported in Supporting Information (S1 and S2 Tables; S1 and S3 Figs).

## Results

**Fig 1:**
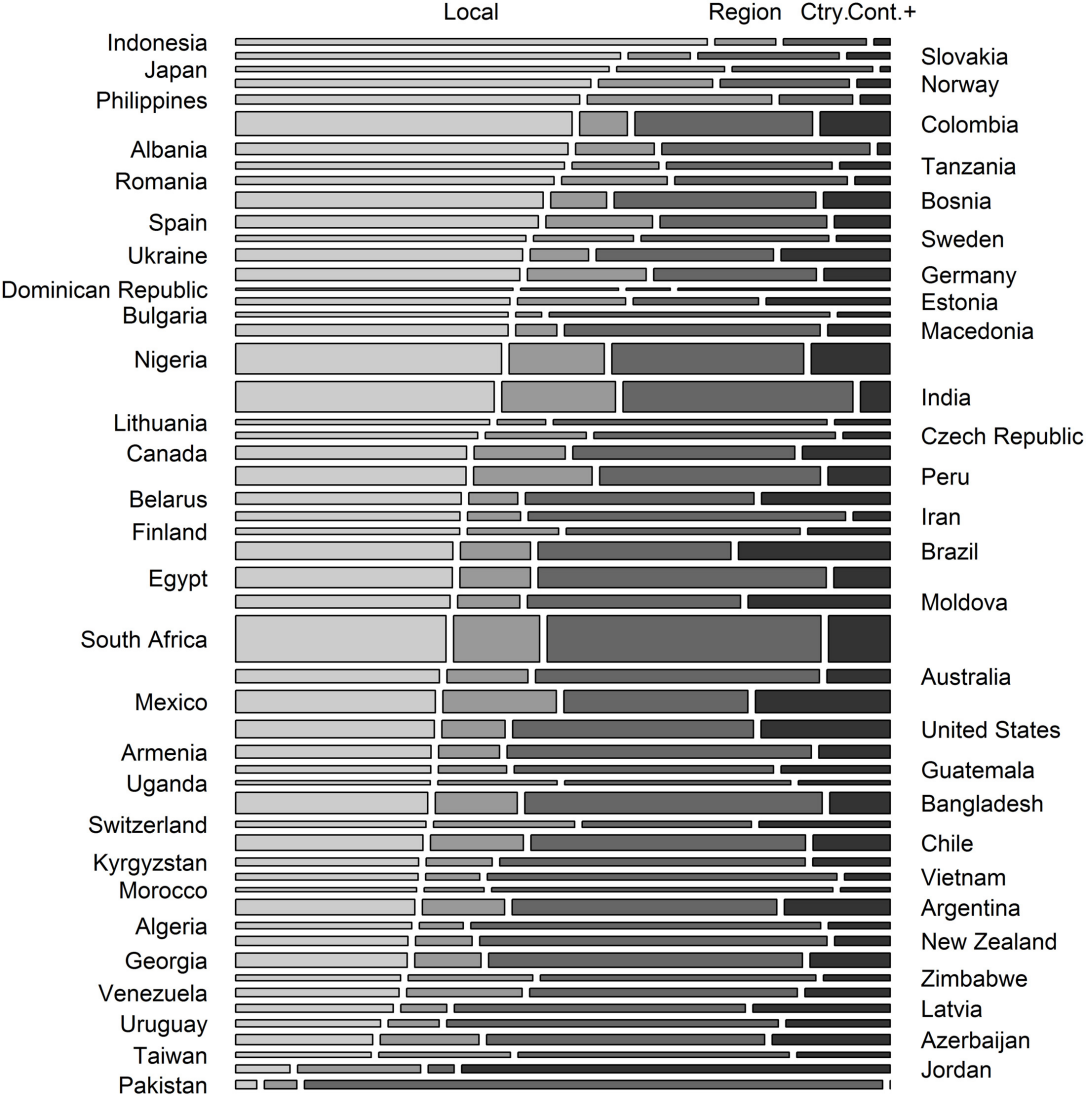
Proportion (box height) of participants with each primary geographic identity (box width) across 55 countries. “Cont.+” represents participants with a continent or world identity.

**Fig 2:**
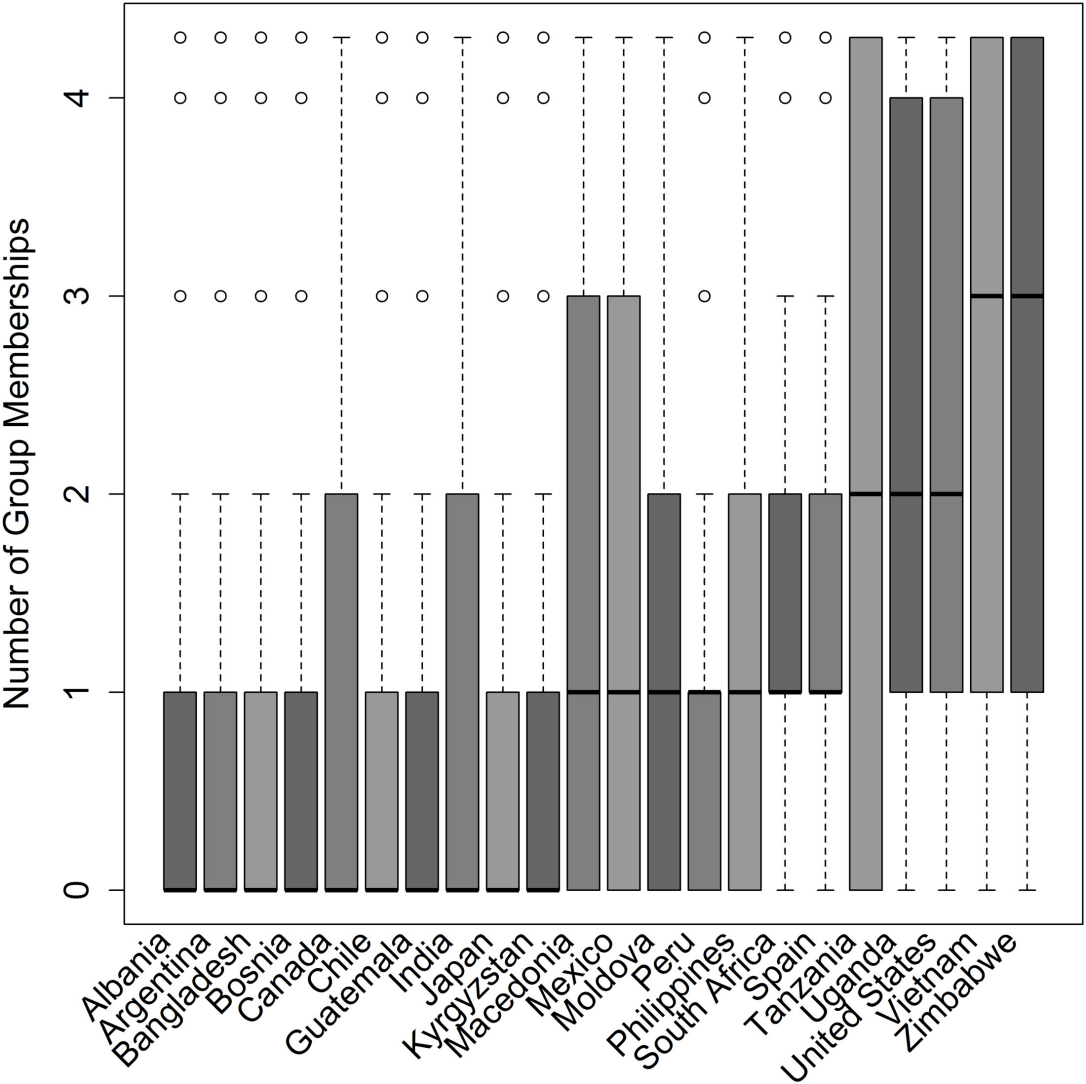
The distribution of participant's number of group identities across 22 countries.

Descriptive statistics appear in Tables 1 and 2. Local- and country-level identities were the most common primary geographic identity across countries (41% and 34% of participants respectively). Participants had a mean membership in 1.01 (SD = 1.69) groups. Figs 1 and 2 illustrate participants' primary geographic identity (1) and number of group memberships (2) by country. Consistent with findings in the corruption literature, bribery was viewed as more permissible among participants who were male, those who did not believe in God, and those who had lower confidence in police and civil services (Tables 3 and 4).

**Table 1:** Descriptive statistics for continuous variables.

Variable	Mean	Median	SD	Minimum	Maximum	N
***Predictor***						
*Number Group Memberships*	1.01	0	1.69	0	4.31	131499
***Individual-level Variables***						
*Subjective Household Income*	5.79	6	2.60	1	10	271871
*Objective Household Income*	4.67	4	2.42	1	11	277865
*Subjective Health*	3.78	4	0.92	1	5	336387
*Number of Kids*	1.82	2	1.57	0	5.29	299780
*Age*	42.09	40	16.71	14	108	373701
*Confid*. *in Police*, *Civil Service*	3.00	3	1.46	0	6	340846
*Town Population (integer)*	4.76	5	2.52	1	9	272798
***Country-level Variables***						
*Religious Fractionalization*	0.48	0.44	0.26	0.01	0.94	165029
*Religious Polarization*	0.53	0.54	0.21	0.02	0.89	165029
*Ethnic Fractionalization*	0.36	0.32	0.23	0.04	0.93	132331
*Ethnic Polarization*	0.50	0.49	0.23	0.08	0.94	132331
*Average Gini*	3.55	3.52	0.25	2.97	4.21	366968
*CPI*	5.61	6.19	2.38	0.69	10.00	383240
*Political Rights Index*	2.48	2	1.86	1	7	383240
*Log Population Density*	4.32	4.40	1.06	1.87	6.67	372526
*Log Population Size*	16.84	17.04	1.49	13.69	20.07	377153
*Year of Interview*		1999		1981	2009	383240

**Table 2:** Descriptive statistics for categorical variables.

Variable	Level 1	Level 2	Level 3	Level 4	N	Interpretation
***Predictor***						
*Primary Geographic Identity*	0.41	0.14	0.34	0.10	296554	Level 1 = local, level 4 = cont./world
***Individual-level Variables***						
*Highest Education*	0.35	0.44	0.22	---	302548	Level 1 = lowest, level 3 = highest
*I Get What I Want*	0.65	0.35	---	---	51218	Level 1 = no, level 3 = yes
*Believe in God*	0.16	0.84	---	---	275959	Level 1 = no, level 2 = yes
*Participant Sex*	0.48	0.52	---	---	378634	Level 1 = male, level 2 = female

**Table 3:** Primary geographic identity (baseline = local) and corruption permissibility^1^,^2^.

Variable	Odds Ratio	Std. Error	z value	p value
*(Intercept)* ^3^	3.82	0.07	18.81	<0.001
*Region*	1.15	0.03	5.05	<0.001
*Country*	0.89	0.02	-5.27	<0.001
*Cont*.*/World*	0.98	0.03	-0.74	0.46
*Shortfall*	1.02	0.01	3.80	<0.001
*Shortfall* ^2^	0.98	0.00	-10.57	<0.001
*Education Level 2*	0.86	0.02	-6.80	<0.001
*Education Level 3*	0.77	0.03	-9.41	<0.001
*Believes in God*	0.84	0.03	-5.49	<0.001
*Confid*. *Police*, *Govt*. *Srvc*.	0.98	0.01	-3.44	<0.001
*Sex*: *Female*	0.85	0.02	-9.11	<0.001
*Age*	0.98	0.00	-22.99	<0.001
*Number of Kids*	0.97	0.01	-4.19	<0.001

^1^Models with random country intercepts, country-level variables, or town population size (with country fixed effects) provide highly similar results, and so are not reported. Reported model n = 80,390. Country fixed effects not reported.

^2^AIC selection criteria suggest that the model including primary geographic identity provides a better fit than the model with only controls (weighted AIC_in-group size_ = 1; AIC_null_ = 79,565.87, AIC_in-group size_ = 79,489.08).

^3^The intercept represents participants with regional identities, who had the lowest household resource shortfall, the lowest level of education, reported the lowest confidence in police and civil services, did not believe in God, had no children, were 0 years old, and male.

**Table 4:** Number of group memberships and corruption permissibility^1^,^2^.

Variable	Odds Ratio	Std. Error	z value	p value
*(Intercept)*	2.60	0.12	7.93	<0.001
*Number of Memberships*	1.08	0.01	5.86	<0.001
*Shortfall*	1.04	0.01	3.62	<0.001
*Shortfall* ^*2*^	0.98	0.00	-5.75	<0.001
*Education Level 2*	0.80	0.04	-5.44	<0.001
*Education Level 3*	0.71	0.05	-6.53	<0.001
*Believes in God*	0.77	0.07	-3.46	<0.001
*Confid*. *Police*, *Govt*. *Srvc*.	0.97	0.01	-2.85	<0.01
*Sex*: *Female*	0.89	0.03	-3.38	<0.001
*Age*	0.99	0.00	-10.48	<0.001
*Number of Children*	0.98	0.01	-1.38	0.17

^1^Models with random country intercepts, country-level variables, or town population size (with country fixed effects) provide highly similar results, and so are not reported. Reported model n = 23,288.

^2^AIC selection criteria suggest that the model including primary geographic identity provides a better fit than the model with only controls and resource shortfall variables (AIC_in-group size_ = 0.99; AIC_null_ = 22,277.93, AIC_in-group size_ = 22,245.83).

### Primary Geographic Identity (P1.1)

#### Participants who report having a larger primary geographic identity will find someone accepting a bribe to be less permissible

**Fig 3:**
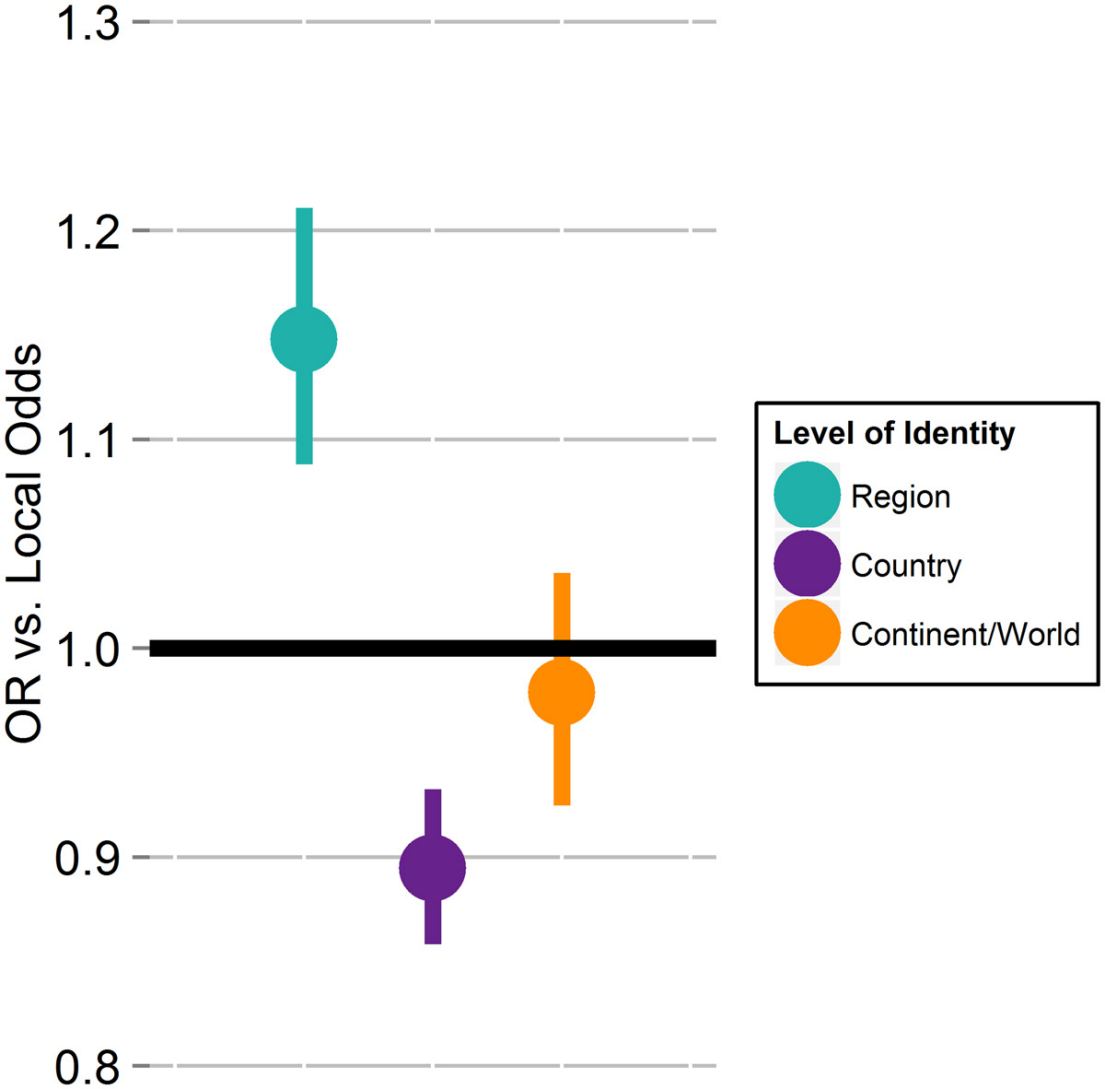
Odds of finding corruption permissible for three levels of primary geographic identity relative to regional identity.

Consistent with P1.1, individuals with a country identity find corruption less permissible than do individuals with a local identity (odds ratio (OR) for country level is 0.89, p<0.001). However, contrary to P1.1, those with a regional identity found corruption more permissible than those with a local identity (OR = 1.15, p<0.001), while those with local and continent or world identities did not differ in their perceived corruption permissibility (continent or world identities OR = 0.98, p = 0.46). The difference in corruption permissibility between those with local and regional identities holds in logistic models with individual-level controls, whether we control for country of residence as a fixed effect or random intercept, include country-level variables instead of country controls, or include a control for the size of the participant’s home town (Table 3; Fig 3; S1 Table; S1 Fig).

To determine whether the pattern of this relationship differs among countries, we regressed corruption permissibility on the individual-level variables separately by country. We held local identity at baseline. The 95% confidence intervals for the ORs for regional, country, or continent or world identities did not overlap OR = 1 in 24 of 55 countries, suggesting that these countries could be driving the effect. Re-running the full model with these 24 countries excluded dampens the magnitude of the contrasts, but does not change the direction of the effect for participants with local or country identities (regional identity OR = 1.07, p = 0.13; country identity OR = 0.96, p = 0.15; continent or world identity, OR = 1.02, p = 0.71; n = 39,328).

For the subset of data for which country-level variables were available, we explored whether different features of a participant’s country of residence could be driving the observed effects. We divided this subset into countries below and above the sample median for Gini coefficient; we then derived parameter estimates separately for countries below the median and for countries above. We repeated this process for absence of political rights (i.e. countries above the median have fewer political rights), perceptions of country-level corruption prevalence, and religious heterogeneity (S2 Fig). Results suggest that the non-linear relationship between primary geographic identity and corruption permissibility may be a product of participants’ responses from countries with high economic inequality, few political rights, low perceived corruption at the country level, and low levels of religious heterogeneity. Two countries, Chile and South Africa, both had few political rights, low country-level corruption, and high Gini; together, they represent 38% of the subset for which country-level variables were available. However, excluding them from analyses does not change the pattern of results, again suggesting that the non-linear pattern is not solely a consequence of country of residence.

### Number of Group Memberships (P1.2)

#### Participants who belong to a greater number of groups will find someone accepting a bribe to be less permissible

Controlling for country and individual-level variables, contrary to P1.2, we find that every additional group a participant belongs to is associated with a 8% *higher* probability of finding corruption permissible (p<0.001), the direction opposite of that predicted (Table 4). This finding is robust to the inclusion of country random intercepts, country-level variables, and the size of the participant’s home town (Table 4, S2 Table, S3 Fig), though the use of world region fixed or random effects in place of country lowers the magnitude of effect (2% higher probability of finding corruption permissible, p = 0.05).

**Fig 4:**
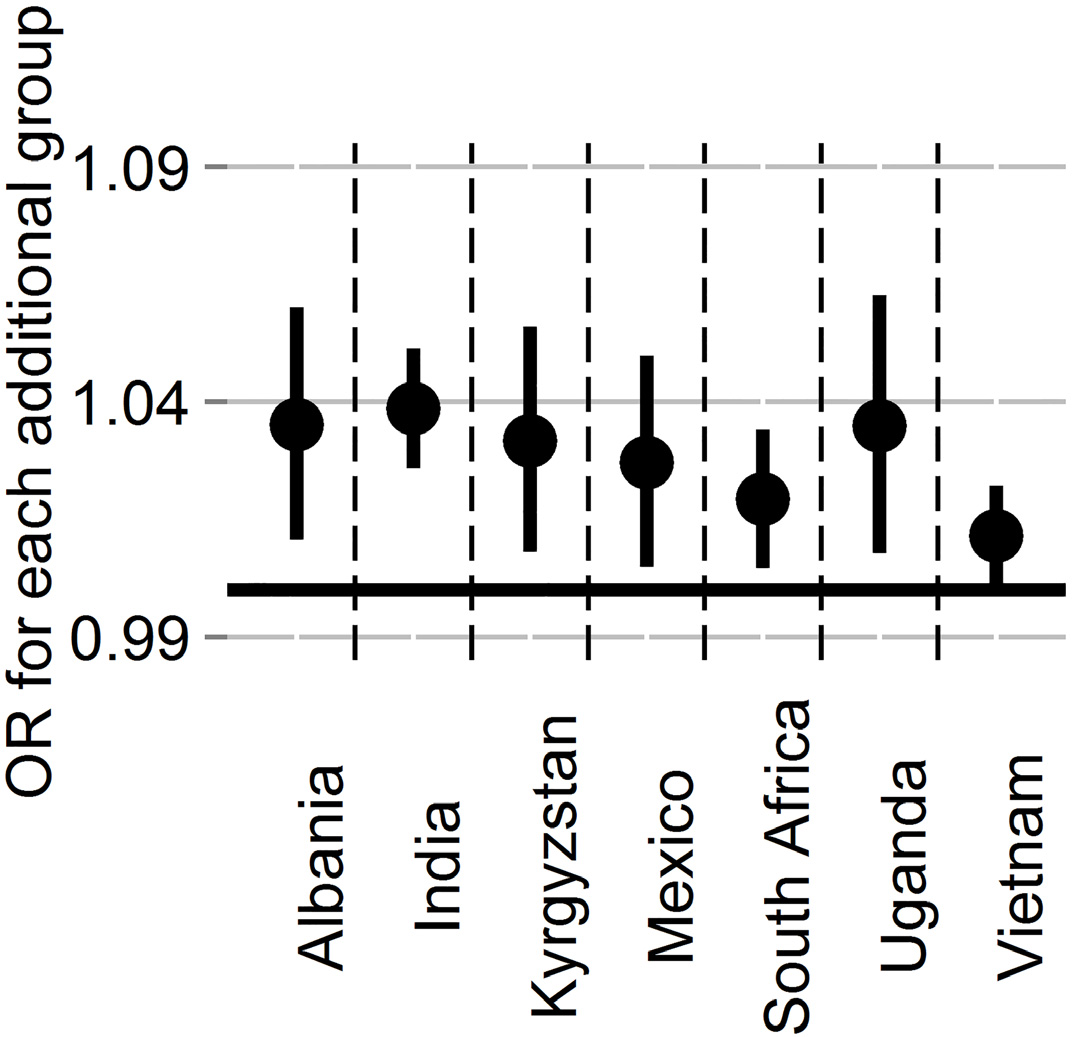
Odds of finding corruption permissible with each additional membership in countries whose 95% CI >0.

Looking at within-country patterns, confidence intervals for the ORs do not overlap OR = 1 in seven of the 22 countries in the sample (Fig 4). Participants in these countries were also likely to be members of more groups than participants in other countries (1.40 vs 1.27, t = 6.34, df = 15,659.05, p<0.001). These countries *do* appear to be driving the effect: once they are removed from the sample, number of group memberships does not predict corruption permissibility in full models with controls (p = 0.78). As only two of these seven countries have the full set of country-level variables, we cannot assess whether country characteristics are driving the effect, however the effect is unrelated to that of world region: only two of the seven countries are from the same region (sub-Saharan Africa) and removal of these two nations from the full sample does not replicate the effect (7% higher odds with each group membership, p<0.001, n = 20,525).

The two proxies for superordinate group size, primary geographic identity and number of group memberships, are not consistently associated with one another: while participants with a continent or world identity belong to 0.19 more groups on average than those with a regional identity (p<0.001), there is no difference in number of memberships between those with local, regional, or country identities. When the two proxies for superordinate group size are included together in the same model predicting corruption permissibility (limiting the size of the subsample to n = 23,090), both geographic identity and group membership variables retain their effect. For each additional group membership, a participant has 8% increased probability of finding corruption permissible, while participants with regional identities have a 1.14 odds (p<0,01) and participants with country identities a 0.93 odds (p = 0.06) of finding corruption permissible relative to participants with local identities; there was no effect of having a continent or world identity for this subset (p = 0.69). AIC weights suggest that, for this smaller subsample, the model including both variables provides a better fit (weighted AIC = 0.99) than models including only number of group memberships plus controls, only primary geographic identity plus controls, or only controls.

### Predicted Determinants of the Net Benefits of Corrupt Behavior

#### Participants who stand to gain benefits from additional resource access (i.e. who have intermediate levels of subjective or objectively measured income, or health) will be less likely to find corruption permissible

**Fig 5:**
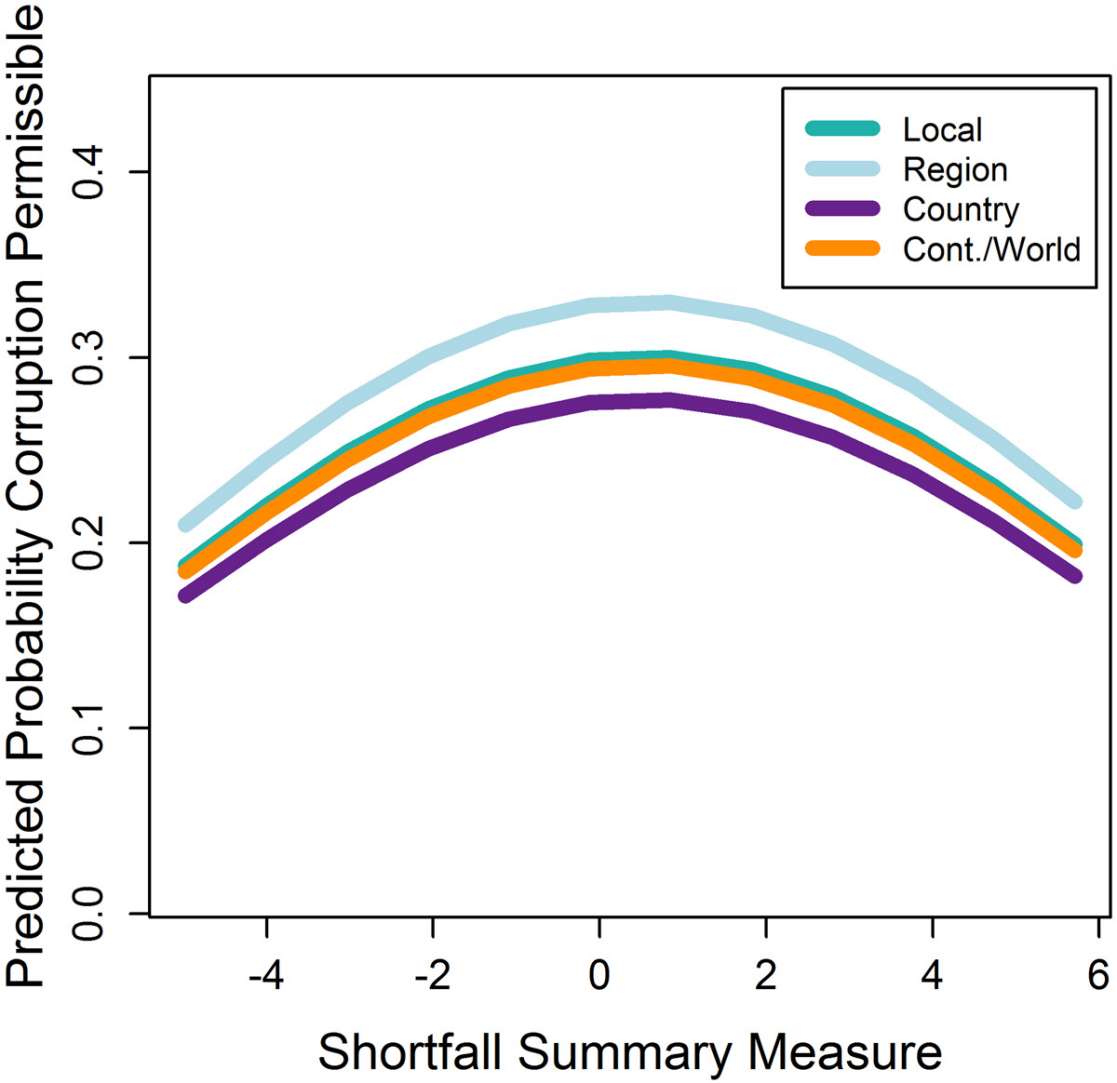
The probability of finding corruption permissible by extent of resource shortfall.

Contrary to P2, participants with intermediate levels of resource availability were significantly more likely to find corruption permissible than those experiencing resource abundance or shortfall (Fig 5; Tables 3 and 4). This relationship was consistent across models.

### Predictors of a Regional Identity: Exploratory Analyses

To explore the consistent, unpredicted relationship between having a regional identity and finding corruption permissible, we tested whether several candidate predictors could account for having a regional identity: kin biases, which have been found to predict corruption previously [1,15] and may trade off with investments in new social partners; profit motive, which has affected tolerance for corrupt behavior in laboratory experiments (e.g. [80]); and respect for authority, which may decrease behavior costly to others [81] and be less common—at least, for a central, national-level authority—in marginalized regions [82]. Across three of four subsamples, participants who believed that greater respect for authority would be a negative change were significantly more likely to have a regional identity than those who believed it would be a positive change (S3 Table). Likewise, believing that greater emphasis on family life would be a *negative* change in one’s life was a positive predictor of having a regional identity across three of the four subsamples.

## Discussion

We hypothesized that the larger an actor’s scope of positively valued actual or potential social partners (i.e. the larger her superordinate identity), the less permissible she would find a corrupt act. We suggested that people have an evolved psychology that enables them to weigh the anticipated benefits of a corrupt act for self, kin, and/or social partners, relative to the anticipated costs of generating harms for bystanders that may include an actor’s positively valued potential social partners. Consistent with this notion, we posited that actors for whom the costs of generating these harms was amplified—for example, those who could benefit from additional resource access and thus may be concerned with maintaining a good reputation—would be less likely to find corruption permissible.

Overall we found mixed support for our hypotheses. Consistent with prediction, we found a relationship between superordinate identity, measured as primary geographic identity, and corruption permissibility. Though more complicated than predicted, this relationship was robust across different model specifications: participants who identified most with their region consistently found corruption more permissible than participants with local or country identities. We did not predict that local identity would be consistently associated with less corruption permissibility than regional identity, primarily because the logic of our argument assumed that harms uniformly affect a broad geographic expanse. To attempt to identify characteristics of participants with regional identities that may drive the relationship between a regional identity and corruption permissibility, we explored whether participants who believed kin to be important, exhibited profit motive, and had a lack of respect for authority were more likely to have regional identities. We found that participants who believed greater emphasis on family life and greater respect for authority would be *negative* changes were more likely to have a regional identity. This analysis was only exploratory, but suggests that actors with regional identities may think of themselves as “free agents,” less concerned about kin, authorities, and the welfare of others—resulting in higher corruption permissibility—than actors with other superordinate identities.

Contrary to our expectations, participants with a larger number of group memberships, our second proxy for in-group size, were *more* likely to find corruption permissible, though this effect was not robust to controls for world region or the exclusion of the seven countries in which the effect was most pronounced. While we expected participants with less access to resources, but not so little access that their time horizon was especially short, to be less likely to find corruption permissible, results suggest that participants with intermediate levels of resource access were actually *the most* likely to find corruption permissible. Previous research has established the importance of the size and exclusivity of groups, as well as the degree of overlap between the groups of which a participant can be a member, for predicting levels of trust. Fukuyama [87] suggests that group number and group size may be inversely correlated: the more groups there are available to an actor, the fewer members are likely to be in each. Thus, belonging to more groups may not actually reflect a larger or broader social community. Groups also vary in the extent to which membership expands generalized trust (cf. [71]). Membership in groups that are open-access is related to generalized trust in the US, whereas membership in restricted access groups is not [88]. The most common groups in our sample were religious groups, labor unions, and sports groups, all of which may have restricted membership. Labor unions and sports teams are also groups that may be competitive in nature: any benefits generated by members may be targeted towards a small set of individuals, potentially at a cost to out-group individuals [89,90]. Additionally, similar to Fukuyama’s argument about how group size may decrease as group number increases, group memberships will also be less predictive of in-group size if groups are highly overlapping [91,92]: the greater the overlap in group memberships (e.g. if members of religious group A are also members of volunteer organization B and political organization C), the less likely generalized trust is to correlate with the number of group memberships [92]. Because of data limitations, we were unable to estimate size, number, or membership constraints of the groups available to an individual. Another possible explanation for the positive relationship between group membership and corruption permissibility is that group membership helps improve household welfare by buffering against resource shortfalls [93], thereby diminishing perceived needs. With a reduced need for resource buffering, one might expect less motivation to build additional social partnerships and less concern about harms from corruption impacting others.

### Present Contribution in Relation to Past Research

Previous research only indirectly addresses whether positive valuation of others affects participation in and tolerance of corruption. In the social capital literature, Fukuyama [87] identifies public corruption as reflecting “a lower standard of moral behavior” towards strangers (p. 9) because “co-operative norms are [not] operative” (p. 8). Warren [94] draws similar conclusions, observing that corruption results from actors targeting reciprocity and trust towards in-group members, generating costs for out-groups who lack the power to counter these harms. Both imply that excluding individuals from cooperation and trust relegates them to out-group status, subject to harms as in-group benefits are generated, but neither connects the withholding of trust and cooperation in the first place to low valuation of these out-group individuals—who may share a superordinate identity with an actor—as potential social partners.

This paper underscores the fact that humans do not value the well-being of a broad scope of other people by default. Some researchers maintain that the human brain can represent relationships with about 150 individuals [95] and that beyond that number, a single value may be used to represent groups of people [52]. Contact with single individuals from out-groups can enable an actor to expand her scope of positive valuation, increasing the size of her superordinate identity [96]. Our results suggest that encouraging a broad scope of positive valuation may curb corruption and tolerance for corrupt acts. For example, anti-corruption campaign materials that personalize those affected by the harms that corruption generates may be more effective than materials that speak of the social ills generated but provide little additional context.

Our present hypotheses that are motivated by evolutionary logic; though these expectations can be derived directly from consideration of how an actor’s valuation of others will affect her decision to engage in corruption, frameworks that consider rational actors as interested in only their own well-being will need modification given evidence of how our evolved preferences impact decision-making. It is unlikely that the mind can calculate *all* benefits and costs of any behavior [97], including corrupt acts. However, a history of natural selection may have favored emotions (and the ability to anticipate the emotions of others) that are sensitive to situational cues, such as those suggesting shared identity or common purpose with others [52,56]. These emotions can therefore motivate actors to consider the welfare of others when engaging in behaviors that confer costs and benefits. For example, contempt for out-group members (and their welfare) [56] might make it easier to support corruption that favors in-groups but harms out-groups, whereas anticipated anger from in-group members who might experience harms [98] could serve to limit corrupt behavior.

Few studies to date have addressed corruption from an evolutionary perspective. Two research groups have modeled how power differentials can lead to elite corruption, both in humans [99] and animals in general [100]. Abdallah and colleagues [101] suggest that centralized punishment, common in state societies, is an evolutionarily unstable strategy for solving public goods provisioning because centralized institutions are susceptible to corruption. Atkinson and Bourrat [72] posit that belief in supernatural punishment makes corruption less permissible, perhaps because "God is watching" and enforcing moral behavior. Newson and Richerson [102] suggest that lowered public corruption encourages state fealty, which may re-direct allegiances from the family and thus contribute to fertility decline.

### Limitations

One limitation of the present study is our inability to examine how survey participants interpret questions about geographic identity, group memberships, generalized trust, and corruption permissibility. To our knowledge, the extent to which survey responses predict propensity to engage in corrupt acts has not been demonstrated. Another open question is how salient and consistent different geographic identities are across countries. For example, the salience of a regional identity may be a function of the political environment [82] and whether people self-select into living in a region because they like it or cannot afford to live elsewhere [103]. Country-specific categories of identity would better capture the relationship between in-group size and corruption permissibility. While WVS data from some countries (such as Colombia) included self-professed membership in different ethnic categories, these were available only for a minority of countries in the WVS and, as we described above, ethnic category membership may not tell us the scope of an actor’s positive valuation. As aforementioned, because of data limitations, we also could not explore who participants thought might be affected by the acceptance of a bribe. It is possible that variable interpretations may underlie some of our reported findings. For example, if participants who identify with marginalized regions interpret the harms generated by corrupt acts as affecting the larger nation, corruption might then be viewed with greater permissibility. However, the permissibility of bribery is likely a conservative proxy of corruption permissibility, as an act such as embezzlement can result in more far-reaching costs. Because of this, we expect the relationship between superordinate identity and corruption permissibility would only increase in magnitude if we adopted a different proxy for corruption permissibility.

As is always the case with cross-sectional survey data, we cannot infer the direction of causality in any of the models presented here. One possible explanation of our results is that past experiences of corruption can both lower perceived corruption permissibility and decrease the scope of positive valuation to the regional level by providing actors with evidence for how others value them. Alternatively, past experiences of corruption may signal to actors that corrupt behavior is status quo, inducing actors to also switch to defection [104], or make an actor feel they are unlikely to caught in a corrupt act [1,21,105]. We attempted to control for past exposures to corrupt behavior by including country-level measures from the Corruption Perceptions Index, but we cannot rule out these alternative explanations.

Because of the necessarily limited scope of the data collected by the WVS, we used country-level data to control for common correlates of corruption—like religious heterogeneity and socioeconomic inequality—which may have better predictive power at a finer grain of geographic scope. Ethnic and religious heterogeneity often differ between different regions of a country (e.g. [106]), as does inequality. The patterns of interaction affected by group membership and inequality on the smaller scale can affect the transmission of cultural norms, which could affect an actor’s willingness to be more tolerant of or generous to strangers at larger geographic scales [107,108]. Further, individual-level experiences of heterogeneity depend on the context, such as the way heterogeneity appears in practice (e.g. markets, policy; [16,88,109]). The same is true for socioeconomic inequality (e.g. [110]). While we were able to include a measure of participants’ subjective socioeconomic status in our models, we were not able to include a measure of a participant’s subjective perception of heterogeneity.

### Future Directions

Our results call attention to the predictive power of superordinate group size for corruption permissibility, independent of other established factors. Further work is needed to elucidate the determinants of a larger scope of positive valuation and its relationship to the perceived benefits and costs of a given action for an actor, including perceived harms affecting bystanders and perceived effects on reputation. Though social scientists have extensive data on the nature of repeated interactions, they have only recently given attention to social identity as a trait that impacts behavior in ways inconsistent with rational choice frameworks (e.g. [111–114]), including how identity affects initial cooperation (e.g. [115–117]). Individual-level data will be crucial for studying these patterns, though much of the existing corruption literature focuses on country-level analyses. Theoretical modeling, ethnographic study, and experiments will be necessary next steps to further explore how an increased scope of positive valuation changes the costs and benefits of an action for an actor.

## Supporting Information

S1 Appendix Country-level variables and statistical methods for country-level variables. (DOCX)

S1 Fig ORs for regional, country, and continent/world identities vs. local identities across models with country-level variables. Either fractionalization or polarization were used as measures of religious and ethnic heterogeneity. For Ethnic & Religious Fractionalization and Religious & Ethnic Polarization subsamples, n = 20,521; Religious Fractionalization and Religious Polarization subsamples, n = 36,997. Ethnic Fractionalization and Polarization subsamples, n = 30,597.
(DOCX)

S2 Fig ORs for countries below and above the sample median for several country-level variables. (Fig A) Gini coefficient, (Fig B) absence of political rights (i.e. above the median means fewer political rights), (Fig C) perceived corruption prevalence, and (Fig D) religious fractionalization. Analyses use the religious & ethnic heterogeneity subsample.
(DOCX)

S3 Fig OR with each additional group membership across models with country-level variables. For the Religious & Ethnic Fractionalization and Religious & Ethnic Polarization subsets, n = 5734; Religious Fractionalization and Religious Polarization subsets, n = 10,874; Ethnic Fractionalization and Ethnic Polarization subsets, n = 8562.
(DOCX)

S1 Table Odds of finding corruption permissible by primary geographic identity for the religious-ethnic heterogeneity subset^1,2,3^. (DOCX)

S2 Table Odds of finding corruption permissible by number of group memberships for the religious-ethnic heterogeneity subset^1,2^. (DOCX)

S3 Table Exploratory analysis regressing having a regional identity (i.e., a binary outcome, where 1 is a regional identity and 0 is a different geographic identity) on participant’s perceptions on whether more emphasis on “family life,” less emphasis on “money and material possessions,” and more emphasis on “greater respect for authority” would be good, neutral, or bad changes; the first and third hold badness at zero, the second goodness. (DOCX)
