# Supplementary material for: Corruption and the Other(s): Scope of Superordinate Identity Matters for Corruption Permissibility
Source: PLoS One. 2015 Dec 9;10(12):e0144542. doi: 10.1371/journal.pone.0144542 (PMC4674100; doi:10.1371/journal.pone.0144542)
Supplement: S1 Appendix — (DOCX) [file pone.0144542.s001.docx]

**S1 Appendix. Country-level variables and statistical methods for country-level variables.**

*1 Country-Level Controls*

*1.1* *United Nations Ethnic and Religious Census Data*

As a measure of objective, country-level religious and ethnic heterogeneity, we employ census data compiled by the United Nations (<http://unstats.un.org/unsd/demographic/sconcerns/popchar/popchar2.htm>). Subjective, individual-level perceptions of heterogeneity were not available from WVS (see Discussion).

To capture the diversity of religions in a given country, we used two methods. First, we adopt the fractionalization index used by Mauro [1], as well as many subsequent authors [e.g. 2,3]. The equation for religious fractionalization is

$${Fractionalization}_{j}=1-\sum_{i=1}^{N} s_{ij}^{2}$$

where *s_ij_* represents the relative share of group *i* (*i*=1, …,*N*) in country *j*. A fractionalization measure of 0 represents maximal homogeneity, while a measure of 1 indicates maximal diversity. We calculated religious fractionalization separately from ethnic fractionalization as the two generate different predictions about government institutions [e.g. ethnic fractionalization predicts less democracy, whereas religious fractionalization may predict more; 2]. Second, polarization, the degree to which people in a country are distributed equally across multiple groups, predicts levels of conflict [4] and failures of collective action [5], whereas fractionalization by itself does not; this difference may be because polarization reflects discrimination by the dominant group [e.g. 5]. To capture this additional dimension of heterogeneity, we also use the polarization index introduced by Montalvo and Reynol-Querol [4]:

$${Polarization}_{j}=1-\sum_{i=1}^{N} \left( \frac{\frac{1}{2}-s_{ij}}{\frac{1}{2}} \right)^{2}s_{ij}$$

where *s_ij_* again represents the relative share of group *i* (*i*=1, …,*N*) in country *j*. A polarization value of 0 represents an even distribution of individuals across *N>>2* groups, and a value of 1 represents the extreme case in which half the population is in one group and the other half in a second group. We again calculated religious and ethnic polarization separately. Although conceptually distinct, the fractionalization and polarization indices are not independent of each other, thus we do not include both in the same model at the same time.

We matched participants’ interview data to religious and ethnic heterogeneity information for their country in the year closest to the year of interview. We report results for the subset of participants from countries for which both ethnic and religious heterogeneity data were available (the "religious-ethnic heterogeneity subset"; only 21 of 37 countries reporting religious heterogeneity data also reported ethnic heterogeneity) separately from models including all participants from countries with religious data and models including all participants from countries with ethnic data.

*1.2 World Bank Population Density, Population Size, Gini Index, and World Region Classifications*

Because both measures of an expanded in-group, primary geographic identity and number of group memberships, will capture a greater number of in-group members in larger countries, we include country population size as an additional control. Indeed, population density is also potentially important, as opportunities for exposure and interactions with a larger number of people may be more common in more densely living populations. We used both population size and density from the World Bank (<http://data.worldbank.org/indicator>) matched to interview year for each country. To minimize the effects of extreme values, we binned values of population size and density above and below 2 SD. We further logged density to minimize the influence of large values, as the distribution of densities was negatively skewed.

To control for economic inequality, a predictor of high levels of corruption [6], we include the World Bank's calculation of country-level Gini index, reported on a 0-100 scale. Because Gini data are only occasionally measured in most countries, we calculated the average Gini across the five years leading up to the year of the interview. To avoid the undue influence of several countries with high inequality, we logged Gini in all analyses.

To test the possibility of that shared cultural history causes correlations in responses between countries, we also use the World Bank’s classification of countries by regions in some models.

*1.3 Freedom House's Freedom in the World Political Rights Index*

Freedom in the World (FIW), a report published by Freedom House that includes indices of political and civil rights, measures the presence of democratic practices in a country (<http://www.freedomhouse.org/report-types/freedom-world>). Government accountability may sway a participant's perspective on corruption permissibility. Because more political rights predict less corruption prevalence at the country level [7] and because of collinearity between the political rights and civil rights indices (r = 0.94), we use only the political rights index. Analysts employed by Freedom House are responsible for assigning countries a score on the political rights index. These scores are based on the following categories: the extent to which citizens in a country can (1) vote as they wish in legitimate elections, (2) run for public office, (3) freely become members of political parties, and (4) vote for accountable representatives. A country can score from 1 to 7, where a country scoring 1 has many political rights and a country scoring 7 has almost none. We matched a participant's interview year with political rights data from the same year.

*1.4 Transparency International's Corruption Perceptions Index*

Researchers commonly model country-level corruption using Transparency International's Corruption Perceptions Index [CPI; e.g. 7,8,9]. Transparency International has published the CPI annually since 1995 (<http://archive.transparency.org/policy_research/surveys_indices/cpi/previous_cpi>). The CPI reflects perceptions of bribery, embezzlement, and kickbacks in the public sector of a given country. The score a country receives is an aggregate of independent surveys of businesspeople, international experts, and risk analysts. This score can range from 0 (very corrupt) to 10 (very clean); we reverse-coded the CPI such that corrupt countries received a 10 to better match the scoring system of FIW. The CPI addresses only two of the four components of the corruption complex, public corruption and embezzlement, but it does provide a control for the rates of public corruption at the country level, which can increase or lower perceived corruption permissibility, as well as obfuscate corrupt acts committed by an individual.

We matched participants with CPI data from the year closest to the year of their interview. Because the CPI began in 1995, this would result in a fairly wide gap for individuals who took the WVS in 1981; however, only 12% of participants in the religious-ethnic heterogeneity subsample were interviewed before 1995, and none before 1990, limiting the size of the gap. Further, we believe the value of the CPI's inclusion outweighs this limitation, given the importance of controlling for country-level corruption prevalence.

*2 Statistical Methods for Country-Level Variables*

Initial analyses revealed high levels of correlation between the seven country-level variables – religious fractionalization, ethnic fractionalization, Gini, population density, population size, the political rights index, and the CPI – and consequent collinearity problems when all were included in the same model. The same was true for religious and ethnic polarization together with the other five variables. To avoid collinearity, we performed a principal components analysis on the seven variables – first with religious and ethnic fractionalization, then with religious and ethnic polarization – for each subset of data. To ensure we summarized at least 80% of the variation in these measures, we extracted the first three components for use in our models. Considering only data from countries for which both religious and ethnic fractionalization data were available, the three components summarize 39%, 27%, and 19% of the variation for the geographic identity subset and 65%, 26%, and 6% for the group membership subset.

**References**

1. Mauro P. Corruption and Growth. Q J Econ. 1995;110: 681–712.

2. Alesina A, Devleeschauwer A, Easterly W, Wacziarg R. Fractionalization. J Econ Growth. 2003;8: 155–194. doi:10.1023/A

3. Easterly W, Levine R. Africa’s growth tragedy: Policies and ethnic divisions. Q J Econ. 1997;112: 1203–1250.

4. Montalvo JG, Reynal-Querol M. Ethnic polarization, potential conflict, and civil wars. Am Econ Rev. 2005;95: 796–816.

5. Waring TM, Bell A V. Ethnic dominance damages cooperation more than ethnic diversity: results from multi-ethnic field experiments in India. Evol Hum Behav. Elsevier Inc.; 2013;34: 398–404. doi:10.1016/j.evolhumbehav.2013.07.003

6. You J, Khagram S. A Comparative Study of Inequality and Corruption. Am Sociol Rev. 2005;70: 136–157.

7. Treisman D. What Have We Learned About the Causes of Corruption from Ten Years of Cross-National Empirical Research? Annu Rev Polit Sci. 2007;10: 211–244. doi:10.1146/annurev.polisci.10.081205.095418

8. Uslaner EM. Producing and consuming trust. Polit Sci Q. 2000;115: 569–590.

9. Schweitzer H. Corruption – its spread and decline. In: Lambsdorff JG, Taube M, Schramm M, editors. The New Institutional Economics of Corruption. London: Routledge; 2005.
