# Supplementary material for: Corruption and the Other(s): Scope of Superordinate Identity Matters for Corruption Permissibility
Source: PLoS One. 2015 Dec 9;10(12):e0144542. doi: 10.1371/journal.pone.0144542 (PMC4674100; doi:10.1371/journal.pone.0144542)

**S1 Figure.** **ORs for regional, country, and continent/world identities vs. local identities across models with country-level variables.** Either fractionalization or polarization were used as measures of religious and ethnic heterogeneity. For Ethnic & Religious Fractionalization and Religious & Ethnic Polarization subsamples, n = 20,521; Religious Fractionalization and Religious Polarization subsamples, n = 36,997; Ethnic Fractionalization and Polarization subsamples, n = 30,597.


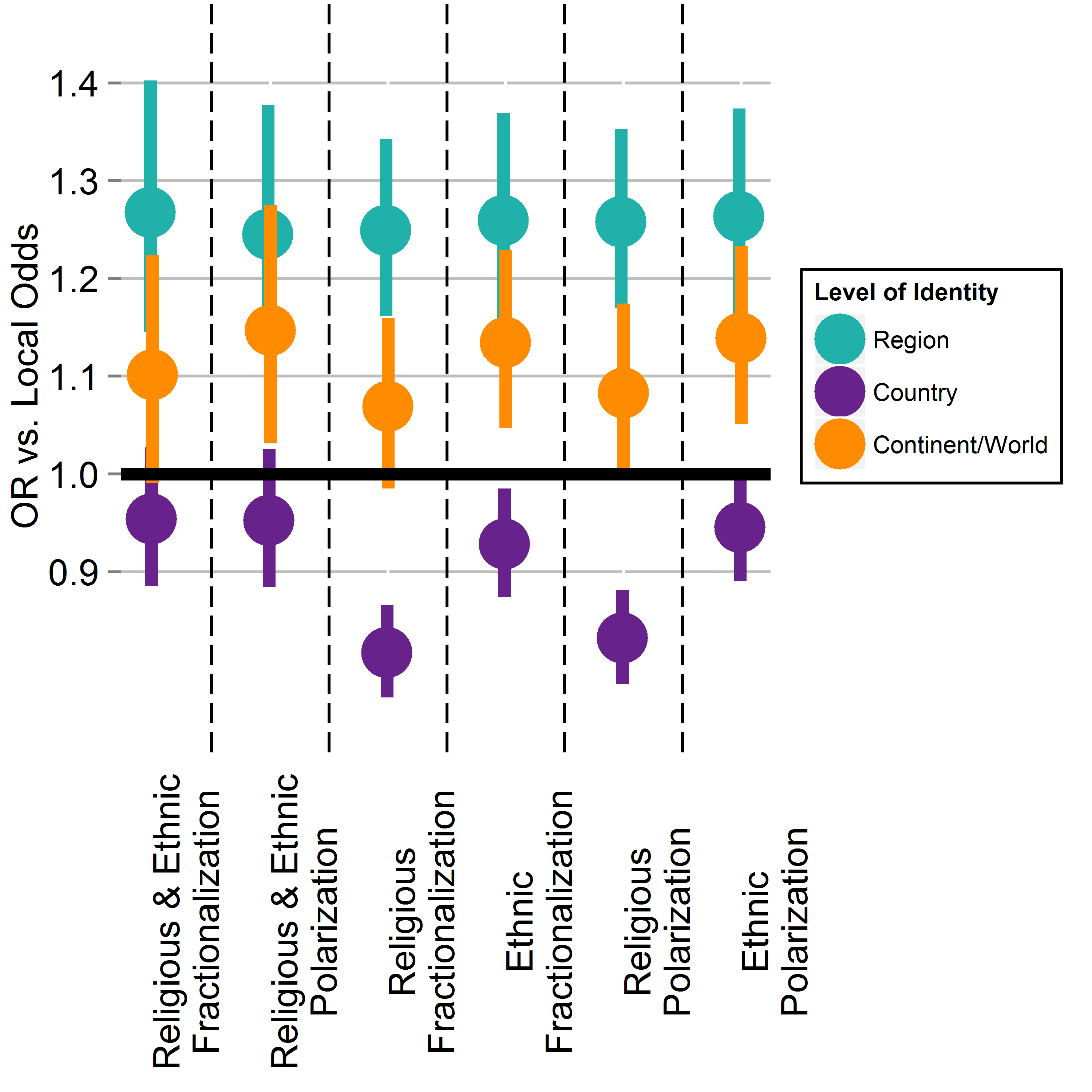

Supplement: S1 Fig — Either fractionalization or polarization were used as measures of religious and ethnic heterogeneity. For Ethnic & Religious Fractionalization and Religious & Ethnic Polarization subsamples, n = 20,521; Religious Fractionalization and Religious Polarization subsamples, n = 36,997. Ethnic Fractionalization and Polarization subsamples, n = 30,597. (DOCX) [file pone.0144542.s002.docx]
