# Supplementary material for: Corruption and the Other(s): Scope of Superordinate Identity Matters for Corruption Permissibility
Source: PLoS One. 2015 Dec 9;10(12):e0144542. doi: 10.1371/journal.pone.0144542 (PMC4674100; doi:10.1371/journal.pone.0144542)

**S2 Figure. ORs for countries below and above the sample median for several country-level variables.** (A) Gini coefficient, (B) absence of political rights (i.e. above the median means fewer political rights), (C) perceived corruption prevalence, and (D) religious fractionalization. Analyses use the religious & ethnic heterogeneity subsample.
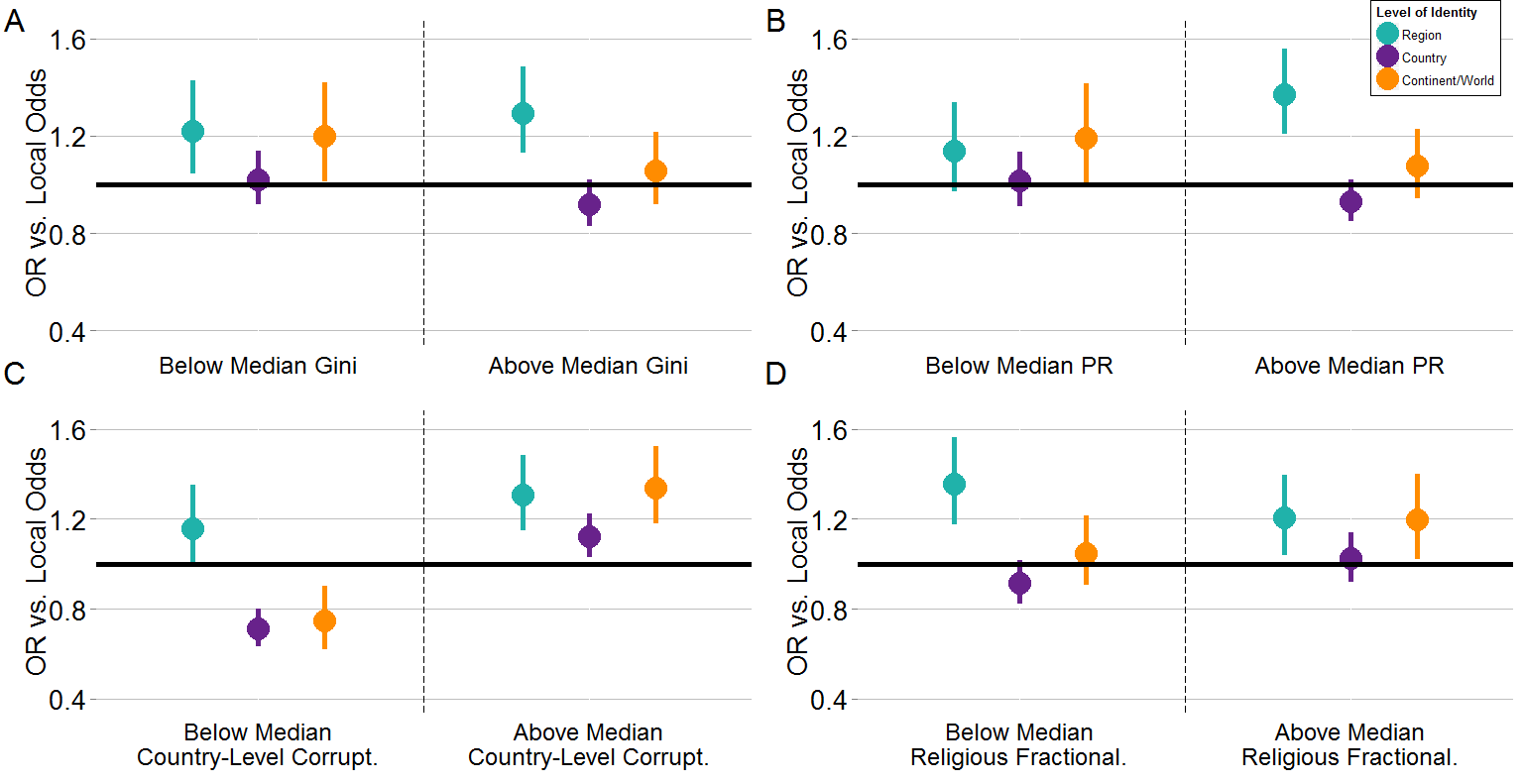

Supplement: S2 Fig — (Fig A) Gini coefficient, (Fig B) absence of political rights (i.e. above the median means fewer political rights), (Fig C) perceived corruption prevalence, and (Fig D) religious fractionalization. Analyses use the religious & ethnic heterogeneity subsample. (DOCX) [file pone.0144542.s003.docx]
