# Supplementary material for: Corruption and the Other(s): Scope of Superordinate Identity Matters for Corruption Permissibility
Source: PLoS One. 2015 Dec 9;10(12):e0144542. doi: 10.1371/journal.pone.0144542 (PMC4674100; doi:10.1371/journal.pone.0144542)

**S3 Figure. OR with each additional group membership across models with country-level variables.** For the Religious & Ethnic Fractionalization and Religious & Ethnic Polarization subsets, n = 5734; Religious Fractionalization and Religious Polarization subsets, n = 10,874; Ethnic Fractionalization and Ethnic Polarization subsets, n = 8562.


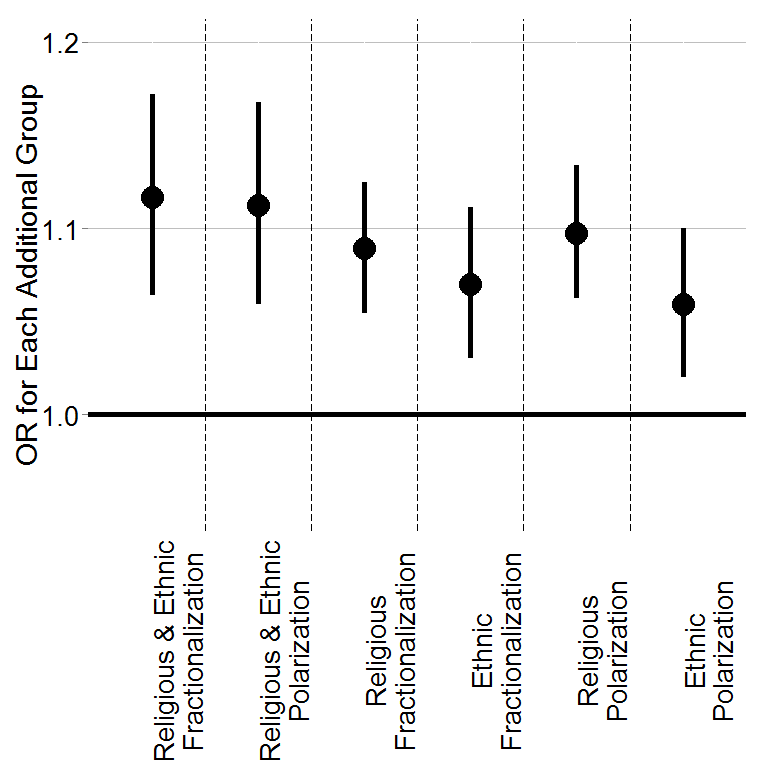

Supplement: S3 Fig — For the Religious & Ethnic Fractionalization and Religious & Ethnic Polarization subsets, n = 5734; Religious Fractionalization and Religious Polarization subsets, n = 10,874; Ethnic Fractionalization and Ethnic Polarization subsets, n = 8562. (DOCX) [file pone.0144542.s004.docx]
