# Supplementary material for: Corruption and the Other(s): Scope of Superordinate Identity Matters for Corruption Permissibility
Source: PLoS One. 2015 Dec 9;10(12):e0144542. doi: 10.1371/journal.pone.0144542 (PMC4674100; doi:10.1371/journal.pone.0144542)
Supplement: S2 Table — (DOCX) [file pone.0144542.s006.docx]

**S2 Table. Odds of finding corruption permissible by number of group memberships for the religious-ethnic heterogeneity subset^1,2^.**

| **Variable** | **Odds Ratio** | **Std. Error** | **z value** | **p value** |
| --- | --- | --- | --- | --- |
| *(Intercept)* | 1.01 | 0.21 | 0.03 | 0.97 |
| *Number of Memberships* | 1.10 | 0.02 | 4.10 | <0.001 |
| *Shortfall* | 1.07 | 0.02 | 3.77 | <0.001 |
| *Shortfall^2^* | 0.98 | 0.01 | -3.64 | <0.001 |
| *Education Level 2* | 0.73 | 0.08 | -3.92 | <0.001 |
| *Education Level 3* | 0.56 | 0.13 | -4.57 | <0.001 |
| *Believes in God* | 0.74 | 0.16 | -1.84 | 0.07 |
| *Confid. Police, Govt. Srvc.* | 0.93 | 0.02 | -3.14 | <0.01 |
| *Sex: Female* | 1.02 | 0.06 | 0.32 | 0.75 |
| *Age* | 0.99 | 0.00 | -3.45 | <0.001 |
| *Number of Children* | 0.95 | 0.03 | -2.19 | 0.03 |
| *Country PC1^3^* | 0.81 | 0.02 | -13.01 | <0.001 |
| *Country PC2* | 1.12 | 0.03 | 4.65 | <0.001 |
| *Country PC3* | 1.23 | 0.05 | 4.20 | <0.001 |

^1^Models with random country or continent intercepts, religious fractionalization or polarization models, and ethnic fractionalization or polarization models show very similar results, and so are not reported. Religious and ethnic fractionalization (reported) model n = 5785. Religious and ethnic fractionalization AIC = 6222.30. Religious and ethnic polarization AIC = 6227.49.

^2^AIC selection criteria suggest that the model including primary geographic identity provides a better fit than the model with only controls and the resource shortfall summary measure (AIC_marginal_ = 1; AIC_marginal_ = 6222.30, AIC_null_ = 6235.74).

^3^The higher the value of the first country-level principal component (PC1), the more a participant’s home country has a small but dense population, has a low Gini and low religious fractionalization, but high ethnic fractionalization, few political rights, and high corruption. The higher the value for the second (PC2), the more her country has a big, dense population with political rights, but high Gini, corruption, religious and ethnic fractionalization. The higher the value for the third principal component (PC3), the bigger her country, the lower its religious but higher its ethnic fractionalization, the lower its corruption, the higher its Gini, and the fewer its political rights.
