# Supplementary material for: Corruption and the Other(s): Scope of Superordinate Identity Matters for Corruption Permissibility
Source: PLoS One. 2015 Dec 9;10(12):e0144542. doi: 10.1371/journal.pone.0144542 (PMC4674100; doi:10.1371/journal.pone.0144542)
Supplement: S3 Table — (DOCX) [file pone.0144542.s007.docx]

**S3 Table.** Exploratory analysis regressing having a regional identity (i.e., a binary outcome, where 1 is a regional identity and 0 is a different geographic identity) on participant’s perceptions on whether more emphasis on “family life,” less emphasis on “money and material possessions,” and more emphasis on “greater respect for authority” would be good, neutral, or bad changes; the first and third hold badness at zero, the second goodness.

|  | **Geog. only^1^** | | **Geog. & group^2^** | | **Country-level vars., geog. only^3^** | | **Country-level vars., geog. & group^4^** | |
| --- | --- | --- | --- | --- | --- | --- | --- | --- |
| **Variable** | **Odds ratio** | **p value** | **Odds ratio** | **p value** | **Odds ratio** | **p value** | **Odds ratio** | **p value** |
| *(Intercept)* | 0.23 | 0.00 | 0.26 | 0.00 | 0.17 | 0.00 | 0.19 | 0.00 |
| *Family Emph: Neutral* | 0.90 | 0.17 | 0.99 | 0.94 | 0.89 | 0.46 | 0.94 | 0.83 |
| *Family Emph: Good* | 0.74 | 0.00 | 0.73 | 0.04 | 0.73 | 0.02 | 0.66 | 0.11 |
| *Profit Motive: Neutral* | 0.94 | 0.08 | 0.94 | 0.32 | 0.87 | 0.03 | 0.94 | 0.61 |
| *Profit Motive: Bad* | 0.97 | 0.25 | 0.94 | 0.21 | 0.81 | 0.00 | 0.80 | 0.04 |
| *Authority: Neutral* | 0.93 | 0.10 | 0.79 | 0.00 | 0.89 | 0.22 | 0.61 | 0.01 |
| *Authority: Good* | 0.92 | 0.04 | 0.76 | 0.00 | 0.89 | 0.19 | 0.62 | 0.01 |
| *Sex: Female* | 1.01 | 0.50 | 0.99 | 0.76 | 1.01 | 0.81 | 1.03 | 0.70 |
| *Age* | 1.00 | 0.00 | 0.99 | 0.00 | 1.00 | 0.46 | 0.99 | 0.06 |
| *Number of Kids* | 1.02 | 0.01 | 1.06 | 0.00 | 1.02 | 0.22 | 1.08 | 0.03 |

^1^Subset including the geographic identity predictor: n=77,376; AIC=59,399. ^2^Subset including the geographic identity and group membership predictors: n=22,729, AIC=17,465. ^3^Subset including the geographic identity predictor and country-level variables: n=19,923; AIC=14,288. ^4^Subset including the geographic identity and group membership predictors and country-level variables: n=5683, AIC=3900.
